# Post-Retail Application of Chitosan Coating and UV-C Irradiation: Effects on Strawberry Decay, Fungal Spoilage, and Consumer Acceptance

**DOI:** 10.3390/foods15162851

**Published:** 2026-08-15

**Authors:** Ilana Chapman, Gene Ahlborn, Matthew Arrington, Jeffrey Schachterle, Zachary Hunsaker, Stockton Nelson, Gregory Snow, Milton Consuegra, Jonathan Kershaw

**Affiliations:** 1 Department of Food Science, Brigham Young University, Provo, UT 84602, USA; 2 Department of Plant and Wildlife Sciences, Brigham Young University, Provo, UT 84602, USA; 3 Department of Microbiology and Molecular Biology, Brigham Young University, Provo, UT 84602, USA; 4 Department of Statistics, Brigham Young University, Provo, UT 84602, USA

**Keywords:** chitosan coating, ultraviolet-c, postharvest, food waste, strawberry, decay, consumer acceptance

## Abstract

Food waste is a global issue with significant environmental, economic, and human health impacts. The largest share of household and retail food waste comes from fresh produce, much of which is still edible at the time of disposal. While previous work has investigated shelf-life extension technologies applied to freshly harvested produce, almost no studies have investigated the efficacy of these treatments when applied to post-retail produce. This study investigated the effect of low-dose UV-C exposure and chitosan coating on the quality, microbial load, and visual acceptance of post-retail strawberries. Strawberries were assigned to low-dose UV-C, chitosan dip, or one of three control conditions (no treatment, water dip, or vinegar dip). Strawberries were analyzed for texture, color, weight, decay percent, yeast and mold counts, and fungal presence via qPCR. Consumer acceptance was determined using an online survey with photographs. Chitosan, but not UV-C, delayed decay by approximately 1 day and lowered yeast and mold counts below limits of detection while having minimal impact on texture and color. Chitosan-treated strawberries were more visually accepted by consumers by Day 1 and Day 2, indicated by higher liking scores and greater association with safety and willingness to eat. UV-C exposure at the low fluence applied did not reduce decay or microbial load during storage. Prolonging the acceptability of post-retail produce provides a meaningful target for reducing household and retail food waste.

## 1. Introduction

Food loss and waste result in more than US$1 trillion of economic losses and account for 8–10% of global greenhouse gas emissions. Food waste—which excludes postharvest losses—accounts for 19% of available food in the world; 73% of food waste comes from the household and retail levels [1]. Fresh fruits and vegetables specifically are a highly wasted food, accounting for approximately 43% of all wasted food in the United States [2]. Importantly, edible and safe produce past peak freshness is often discarded because of salability concerns, inventory management constraints, and consumer expectations of visual quality [3]. For consumers, surface blemishes reduce perceived sensory quality and increase the likelihood that fruit will be rejected before it is eaten [4]. Thus, appearance and perceived spoilage—rather than confirmed microbial or chemical safety failure—are directly connected to discard decisions at both the retail and household level [5,6,7]. Delaying spoilage and prolonging the quality of post-retail produce may extend the window of consumer acceptability and result in less wasted food.

Although a range of shelf-life extension technologies have been developed to slow postharvest deterioration, their efficacy in post-retail fruit is largely untested. Established technologies, such as ultraviolet irradiation, edible coatings, and modified atmosphere treatments, have been effective in delaying the decay of produce [8]. Many of these strategies function, at least in part, by eliciting host-plant defense responses, including antioxidant enzyme activity, phytohormone signaling, and phenolic compound synthesis [9,10]. These defense responses, however, are diminished in aging tissue [11,12]. For example, the antioxidant capacity that shelf-life extension treatments often amplify in early-stage fruit may be substantially depleted by mid-to-late post-retail aging [13,14]. Furthermore, surface antimicrobial strategies such as chlorine or salt baths may have limited effectiveness in post-retail produce, as mold has often penetrated beyond the surface level [15]. Thus, post-retail produce may have a different physiological response to shelf-life extension treatments than freshly harvested produce. Relatively few studies have examined the application of shelf-life extension technologies to fruit that has already undergone retail handling and is physiologically aging and entering early spoilage.

Strawberries are an appropriate model for studying post-retail intervention due to their high respiration rates, delicate skin, temperature sensitivity, and susceptibility to fungal decay [16,17,18,19,20]. Strawberries are widely cultivated globally and are frequently identified as a high-waste commodity in food loss research [21,22]. Under appropriate cold-storage conditions, strawberries have a senescence timeline of approximately seven days and typically arrive at retail approximately three days postharvest [23,24]. Post-retail strawberries therefore represent a mid-to-late senescence stage (approximately days 3–5), at which some residual antioxidant capacity may remain, but surface mold colonization has often begun [25,26]. The primary spoilage mechanisms of strawberries are microorganisms, especially the mold *Botrytis cinerea*, which enters through damaged tissue [15,26]. Thus, fungal control is a key target in post-retail quality preservation of strawberries.

UV-C irradiation and chitosan-based edible coatings are two postharvest strategies with documented efficacy in freshly harvested berries [27,28], but whether their protective mechanisms remain effective when applied to post-retail fruit is not established. UV-C irradiation (200–280 nm) is approved by the U.S. Food and Drug Administration for food irradiation and extends produce shelf-life by disrupting microbial DNA and RNA at the fruit surface, causing cell death [27,28,29]. In freshly harvested strawberries, UV-C has also been shown to activate antioxidant defense systems, increase phenolic compound concentrations, and influence abscisic acid and anthocyanin accumulation [28,30,31,32]. Decay control in whole strawberries has been demonstrated at 0.25 kJ/m^2^, while damage may occur at doses above 1.0 kJ/m^2^ [33]. Excessive UV-C doses can cause host cell DNA damage and promote excess antioxidant enzyme activity [34]. Despite the documented efficacy of UV-C on surface contamination, it has low penetration power in solids and requires direct and even exposure [35,36]. The product’s ripening stage has also been identified as a factor that impacts UV-C efficacy [35,37]. Because UV-C’s host-defense benefits depend on active phytohormone signaling, its efficacy may be modulated in tissue where those pathways are already declining, such as post-retail produce. Despite these limitations, the sterilizing effect of UV-C on fruit surfaces may provide some benefits; thus, investigating the efficacy of UV-C treatment in post-retail produce merits further exploration, despite the limitations of ripening stage and UV-C penetration.

Chitosan, derived from chitin found in crustacean shells and fungal cell walls, possesses barrier, antimicrobial, antifungal, and antioxidant properties [38,39]. Fungal-derived chitosan is generally recognized as safe (GRAS) for postharvest fruit coatings [40]. In freshly harvested strawberries, chitosan delays decay through multiple mechanisms, such as reduced moisture and gas transfer, direct antifungal activity against *Botrytis cinerea*, and activation of antioxidant enzymes [13,26,41,42,43,44]. Because chitosan’s direct antifungal activity is less dependent on active host defense signaling than UV-C’s mechanism, chitosan may retain some efficacy in post-retail fruit; however, the effects of chitosan on post-retail quality are largely untested.

Given the contribution of edible, post-retail produce to food waste, the purpose of this study is to evaluate whether UV-C irradiation or chitosan coating could preserve the quality and visual acceptability of post-retail strawberries. In addition to objective measures such as visible decay, microbiological measures, weight loss, color, and texture, the inclusion of visual consumer acceptance data connects quality changes with likely consumer rejection behavior. Exploring whether produce shelf-life extension technologies are effective in post-retail produce in a way that matters for disposal behaviors can provide an additional strategy to help reduce food waste.

## 2. Methods

### 2.1. Sample Collection, Retail Simulation, and Storage

Strawberries (*Fragaria ananassa*) (Gem-Pack Berries, Irvine, CA, USA) were purchased from the same retail location (Smith’s Food and Drug, Provo, UT, USA) for each experimental block at the same time on the day shipments arrived at the store. The inherent variability in upstream handling of the strawberries is acknowledged; however, this variability reflects the real-world conditions faced by consumers and grocery rescue programs, which strengthens the external validity of the study. Strawberry containers were immediately transported and placed into an environmental chamber (Environmental Growth Chamber, Chagrin Falls, OH, USA) in their native closed plastic clamshell containers to simulate conditions in a retail environment (temperature 8 °C ± 2 °C and 60% ± 15% relative humidity RH). The container consisted of a clear, rigid, hinged polyethylene terephthalate (PET) clamshell with multiple ventilation openings distributed across the lid, sidewalls, and base and a stated net weight capacity of 907 g. Temperature and humidity ranges were chosen based on observations at the retail location (measured using a temperature and humidity sensor, YS8003-UC, Yolink, Lake Forest, CA, USA) and were consistent with previous studies of strawberry decay [45,46,47]. Strawberries were held for 24 h total, consistent with standard retail holding times [45,46,47].

After the retail simulation, fruits with visible mold or physical damage were removed, representing approximately 3% of the strawberries in each experimental block. The remaining strawberries were removed from their original retail containers and randomly assigned to treatment groups. Within each treatment group, strawberries were randomly allocated among four of the original clamshell containers, with one container assigned to each of the four sampling days. Each container held 18–25 strawberries and served as the experimental unit for its treatment-by-day combination. Strawberries were then treated according to their assigned treatment, as described below. Following treatment, strawberries were returned to their assigned containers and placed in the environmental chamber at 21 °C ± 2 °C and 60% ± 15% relative humidity to simulate an indoor room temperature environment. Room temperature conditions were chosen to simulate holding conditions in grocery rescue programs, where access to refrigerated storage is limited, thus increasing the ecological validity of the study.

### 2.2. Strawberry Treatment

A total of six experimental blocks were conducted over the course of the study, each corresponding to a single strawberry purchase treated on its own date. Each block was separated by approximately 2–3 weeks. Block-level baseline variation was accounted for by including block as a random effect in the mixed model, as described in the statistical analysis section. Treatments within the first three blocks directly compared the non-treatment control, UV-C, chitosan, and water control. To elucidate whether chitosan effects could be explained by the presence of the acetic acid in the dispersing media, two additional blocks were conducted with only the control, chitosan, and 20% vinegar solution treatments. For clarity of reporting, these five experimental blocks were combined into a single analysis; however, because the statistical analysis only compared treatments conducted within the same block, the most robust comparisons are those between treatments that shared a block (e.g., control vs. chitosan), while cross-block comparisons (e.g., UV-C vs. vinegar) are weaker. To investigate whether treatments altered the fungal population, an exploratory sixth block was conducted with all five treatments where only fungal DNA was quantified, as described below.

Ultraviolet-C Treatment. Strawberries assigned to UV-C treatment were unpackaged and placed in a single layer on a raised wire surface with at least 2 cm space between each fruit inside an enclosed 100.84 cm (length) × 46.48 cm (width) × 43.93 cm (height) opaque plastic bin (Project Source Commander, Mooresville, NC, USA). UV-C exposure was applied using a Spectroline X-15G lamp equipped with a 15 W, 254 nm emitting tube (Spectro-UV, Farmingdale, New York, NY, USA) placed 34 cm above the surface of fruits and moved across the length of the bin for a total of nine seconds. The strawberries were then turned 180 degrees to expose the underside, and the UV-C exposure was repeated. Based on the manufacturer-reported tube intensity of 50.5 µW/cm^2^ at 1 m, irradiance at 34 cm was estimated at approximately 437 µW/cm^2^ using an inverse-square approximation. This corresponds to an estimated UV-C dose of 3.9 mJ/cm^2^ per side and a nominal two-sided process dose of 7.9 mJ/cm^2^. Because irradiance was not directly measured at the fruit surface, this value should be interpreted as an estimated nominal dose. Furthermore, no measures were taken to account for shading effects; thus, variable exposure is a limitation of this study. UV-C exposure time was based on preliminary testing that minimized premature mold growth or skin discoloration. Gloves were changed between each container, and 70% ethanol cleaner was used between each group to limit possible cross-contamination. After treatment, strawberries were returned to sanitized native clamshell containers and stored in the room temperature-controlled environmental chamber (21 °C ± 2 °C, and 60% ± 15% RH).

Chitosan Treatment. Powdered chitosan derived from *Aspergillus niger* (Modernist Pantry LLC, Eliot, ME, USA) was used to make a 1% chitosan solution consisting of 20% distilled white vinegar (5% acetic acid, Kroger Co., Cincinnati, OH, USA) and 80% distilled water. The solution was blended in a glass beaker with an immersion blender until fully homogeneous. Strawberries assigned to the chitosan treatment were removed from their containers and submerged in the chitosan solution for five minutes. Fruits were then removed and allowed to air-dry for 90 min on slightly elevated wire racks [48]. Strawberries were then repackaged into sanitized plastic clamshell containers and stored in the room temperature-controlled environmental chamber.

To better compare the effect of the chitosan treatment alone vs. liquid submersion or the presence of vinegar, both a water treatment and a vinegar treatment were included using the same solution preparation, five-minute submersion, and drying procedures used for the chitosan treatment.

### 2.3. Measurement of Strawberry Quality and Microbial Presence

The day of treatment was designated as Day 0, and measurements were taken at 24 h intervals for up to Day 4 (or 96 h post-treatment). Weight, decay, and fungal population were evaluated beginning on the day of treatment (Day 0), while texture, color, and microbial growth were evaluated beginning on Day 1. Mold and yeast counts were taken from the container before color and texture measurements to prevent cross-contamination. Due to high rates of decay by 96 h post-treatment, texture and yeast and mold counts are not reported.

Weight loss. Weight loss was measured gravimetrically. An empty container was used to tare the scale, and then each strawberry container was measured directly after treatment. Moisture loss was quantified as a percent of initial weight.

Decay percentage. Decay percentage was calculated as the number of strawberries with visible decay, divided by the total number of strawberries in that container, times 100. Visible decay was defined as any noticeable mold growth on the berry, any noticeable soft spots or damage, or both [22].

Color. L*a*b* values were quantified using the Hunterlab ColorFlex EZ (Reston, VA, USA) spectrophotometer. Strawberries (*n* = 15) were placed on the colorimeter and rotated along its stem-to-bottom axis to obtain three readings, which were averaged to produce one observation per fruit.

Texture analysis. Texture was measured using the TA XT Plus C texture analyzer (Texture Technologies Corp, Hamilton, MA, USA). Following color analysis, the same strawberries were evenly cut in half from stem-side to bottom, and one half was used for texture analysis. A reading was taken from the tallest part of the strawberry with a 5 mm plunger. In total, 15 fruits from each treatment group container were tested by the texture analyzer each day. Bioyield (grams of force applied), skin elasticity (mm returned to original position after compression), stiffness (grams of force divided by the time in seconds), and work to penetrate (grams of force applied times the time in seconds) were calculated; each is a common measurement in fruit texture analysis [49,50].

Yeast and Mold Count Tests. Yeast and mold populations were quantified in triplicate using Neogen^®^ Petrifilm^®^ Yeast and Mold Count Plates (Neogen Corporation, Lansing, MI, USA) according to the manufacturer’s instructions. Approximately 3–5 strawberries per container were randomly selected using a random number generator. For each treatment replicate, 45 g of strawberries were aseptically weighed and homogenized in 450 mL of sterile 0.1% peptone water (BD Difco™, Becton Dickinson, Sparks, MD, USA) to obtain a 10^−1^ dilution. Serial dilutions were subsequently prepared in sterile 0.1% peptone water to yield dilutions ranging from 10^−2^ to 10^−6^. To obtain countable plates, dilutions were selected based on the anticipated microbial load of each container. Strawberries from containers exhibiting low levels of visible decay (0–20%) were plated at the 10^−2^, 10^−3^, and 10^−4^ dilutions, whereas strawberries from containers exhibiting higher levels of visible decay (70–89%) were plated at the 10^−4^, 10^−5^, and 10^−6^ dilutions. Two Petrifilm plates were inoculated from each selected dilution and averaged, resulting in six plates per container. One milliliter of each dilution was aseptically applied to the Petrifilm plates, which were then incubated at 28 °C for 55–60 h. Following incubation, yeast and mold colonies were enumerated. Plates containing 30–300 colonies were considered countable and used for microbial enumeration. Plates containing fewer than 30 colonies were considered below the countable range, whereas plates containing more than 300 colonies were recorded as too numerous to count (TNTC). Counts were converted to log_10_ CFU/g prior to statistical analysis.

DNA extraction for qPCR*.* Samples of 30–50 mg were collected from the surface of treated or untreated strawberries on the day of treatment or 3 days post-treatment, and DNA was isolated using a CTAB lysis method [51] as follows. Fruit samples were homogenized in 500 µL of CTAB lysis buffer (2% (*w*/*v*) cetyltrimethylammonium bromide, 100 mM Tris-HCl, 20 mM EDTA, 1.4 M NaCl, 0.5% β-mercaptoethanol, 2% (*w*/*v*) polyvinylpyrrolidone, 10 mM ascorbic acid) and incubated at 60 °C for 30 min. Lysates were extracted twice with 500 µL of chloroform, and the remaining aqueous phase was mixed with ethanol in a 1:3 ratio and incubated at −20 °C for 5 min to allow DNA to precipitate. Precipitates were collected by centrifugation, washed once with 300 µL of 70% ethanol, and dried, and purified DNA was dissolved in 50 µL of pure water.

Fungal quantitation by qPCR. Purified DNA samples were used as templates in quantitative PCR reactions with SYBR green dye. Reactions were conducted using PowerTrack SYBR master mix (Applied Biosystems, Carlsbad, CA, USA). Primers targeting the strawberry (*Fragaria ananassa*) genome (Fa F: TGCTGTTGGAGTCATCAAGAATG; Fa R: TTGGCTGCAGACTTGGTCAC) were used as a normalization control to obtain the relative quantity of fungal DNA compared to strawberry DNA in samples. Fungal DNA was amplified using existing degenerate fungal primers targeting the fungal ITS sequence (FF390: CGATAACGAACGAGACCT; FR1: ANCCATTCAATCGGTANT; refs. [52,53]. qPCR reactions were cycled using a StepOnePlus system (Applied Biosystems, Carlsbad, CA, USA). The ratio of fungal to strawberry DNA was calculated with the following formula: Ratio = 2^(CtS-CtF)^, where C_t_S represents the qPCR C_t_ value for the strawberry amplification, and C_t_F represents the qPCR C_t_ value for the fungal ITS amplicon. Samples were collected for three biological replicates (individual fruits) at each treatment/time point combination. For each fruit, two technical replicates were tested and pooled.

### 2.4. Consumer Acceptance Survey

Visual consumer acceptance of strawberries was assessed using an online survey. A single picture of an open strawberry container from Day 0, Day 1, and Day 2 from the control, UV-C, water, and chitosan treatments was evaluated. Days 3 and 4 were excluded from the analysis due to higher mold presence. Pictures were taken from a top-down view with a white background in a Zkeezm light box (Zkeezm, Bangkok, Thailand) at 40-watt power using a Canon EOS R50 camera equipped with its standard lens. Pictures were taken 40 cm away from the produce with the lens directly above the strawberries. Strawberries were photographed in an open clamshell container.

Participants were recruited from CloudResearch’s Connect platform [54] and provided informed consent prior to beginning the study. Participants who indicated they consumed strawberries at least once per month, passed an attention check question, and provided informed consent (*n* = 101, 51% female, median age = 46) were then presented with the strawberry images in a random order. For each image, participants were asked, “How much do you like the appearance of these strawberries?” and answered using a 9-point hedonic scale [55]. Participants were then asked, “What is your opinion of these strawberries?” and asked to select any of the following terms that applied: tasty, safe to eat, want to eat, fresh, off-color, moldy, spoiled, off-texture, or none of the above. Terms were selected based on previous studies using images to study consumer perception of degrading produce [56]. All procedures were approved by the Institutional Review Board of Brigham Young University (IRB #2026-233).

### 2.5. Statistical Analysis

All statistical analyses for quality and microbiological outcomes were done in R version 4.6.0 using the tidyverse, lme4, lmerTest, glmmTMB, multcomp, car, survival, interval, Icens, and emmeans packages. Models were either a linear mixed model (LMM) or a generalized linear mixed model with a Gamma log link (GLMM). Coefficients from log-transformed LMMs and from GLMMs with a log link were exponentiated and are reported as percent changes. More information on the modeling can be found in Table 1. Log transformations were applied to weight and bioyield to account for the non-constant variance in the residuals. The weight and colors (L*, a*, b*) had no data removed; however, the texture (bioyield, elasticity, penetration, stiffness) had the same four observations removed due to measurement errors or missing information. There were 121 of 925 (13.1%) elasticity measurements where the skin did not rupture within the probe’s range; these were recorded at the 6 mm boundary and analyzed at that value. A censored regression gave the same ordering of treatment slopes and the same significance structure, with effects 13–22% larger, so the estimates reported here are conservative. To allow for block-level heterogeneity, we used a linear mixed model that allows baseline outcome levels to vary across blocks while constraining the effect of day on the outcome to be fixed across all blocks. The same method was applied to each container within each block, as well as for the color analysis, and each strawberry within each container and block. Not all treatments were present in every block: controls and chitosan were included in all five blocks, UV-C and water in blocks 1–3, and vinegar in blocks 4–5. To confirm that treatment effects were not confounded with block, each model was refit with block as a fixed effect, which restricts all treatment comparisons to within-block information. Estimates changed negligibly, and no comparison changed significance status. All main effects were tested via a Type III ANOVA (F statistics with Satterthwaite denominator degrees of freedom for linear mixed models; Wald χ^2^ for generalized linear mixed models). The interaction between day and treatment was tested with a likelihood ratio test comparing nested models. When the interaction was not significant, it was dropped from the model and only main effects are reported. Pairwise comparisons between treatment groups were computed using estimated marginal means (emmeans) with Tukey’s adjustment, as all pairwise treatment comparisons were of interest. For additive models, these were reported only where the treatment main effect was significant; where the interaction was significant, simple main effects of treatment were tested at each day and pairwise comparisons reported for days on which that effect was significant. ANOVA results are reported in Supplemental Table S1.

Yeast and mold count data included many measurements that were too few to count (<30, approximately 50.8% of data points) or too numerous to count (>300, approximately 23% of data points). These responses were treated as interval censored with the intervals based on the minimum and maximum observed values for each result. A permutation test that takes into account the interval censoring was used to compare the treatments. Statistical significance was assessed at α = 0.05.

Fungal populations were compared using a one-way ANOVA followed by Tukey’s post hoc test for all pairwise comparisons at the same time point (Day 0 or Day 3). Due to the exploratory nature of the fungal population data, independent samples *t*-tests were performed to compare the treatment groups to the control. Differences between groups with *p* < 0.05 were considered to be significant.

Differences in appearance liking were determined using SPSS version 31. A repeated measures general linear model was used to assess the effect of day, treatment, and the Day × Treatment interaction. Because the Day x Treatment interaction was significant, simple effects of treatment were examined at each storage day separately using one-way repeated-measures ANOVAs, with pairwise comparisons among treatment conditions (control, UVC, water rinse, and chitosan) adjusted for multiple comparisons using the Bonferroni correction. Differences between attribute endorsement frequency between treatments for each day were determined by conducting a Cochran’s Q analysis followed by Bonferroni-corrected exact McNemar pairwise comparisons of treatments (α = 0.05).

### 2.6. Generative AI Use in Manuscript Preparation

The large language model Claude (Sonnet models 4.6-5 and Opus model 4.8, Anthropic, PBC; claude.ai) was used in the preparation of this manuscript. The tool was used for the following purposes: (1) Literature synthesis: Claude was used to search academic databases (Consensus.app; Scholar Gateway) following the initial author literature review to locate and synthesize additional sources to inform the Introduction and Discussion Sections. (2) Reference verification: The authors used Claude to query academic databases to evaluate the accuracy of citations in the draft. (3) Section drafting: The authors used outlines, writing guides, drafts, and data summaries along with Claude to iteratively improve clarity and draft some sections of the manuscript. (4) Editing and revising: Claude was used to improve grammar and readability, and to help identify errors or inconsistencies in the manuscript. All AI-generated content was reviewed, edited, and verified by the authors, who take full responsibility for the accuracy, integrity, and originality of the work as submitted.

## 3. Results

### 3.1. Decay Percentage

**Figure 1:**
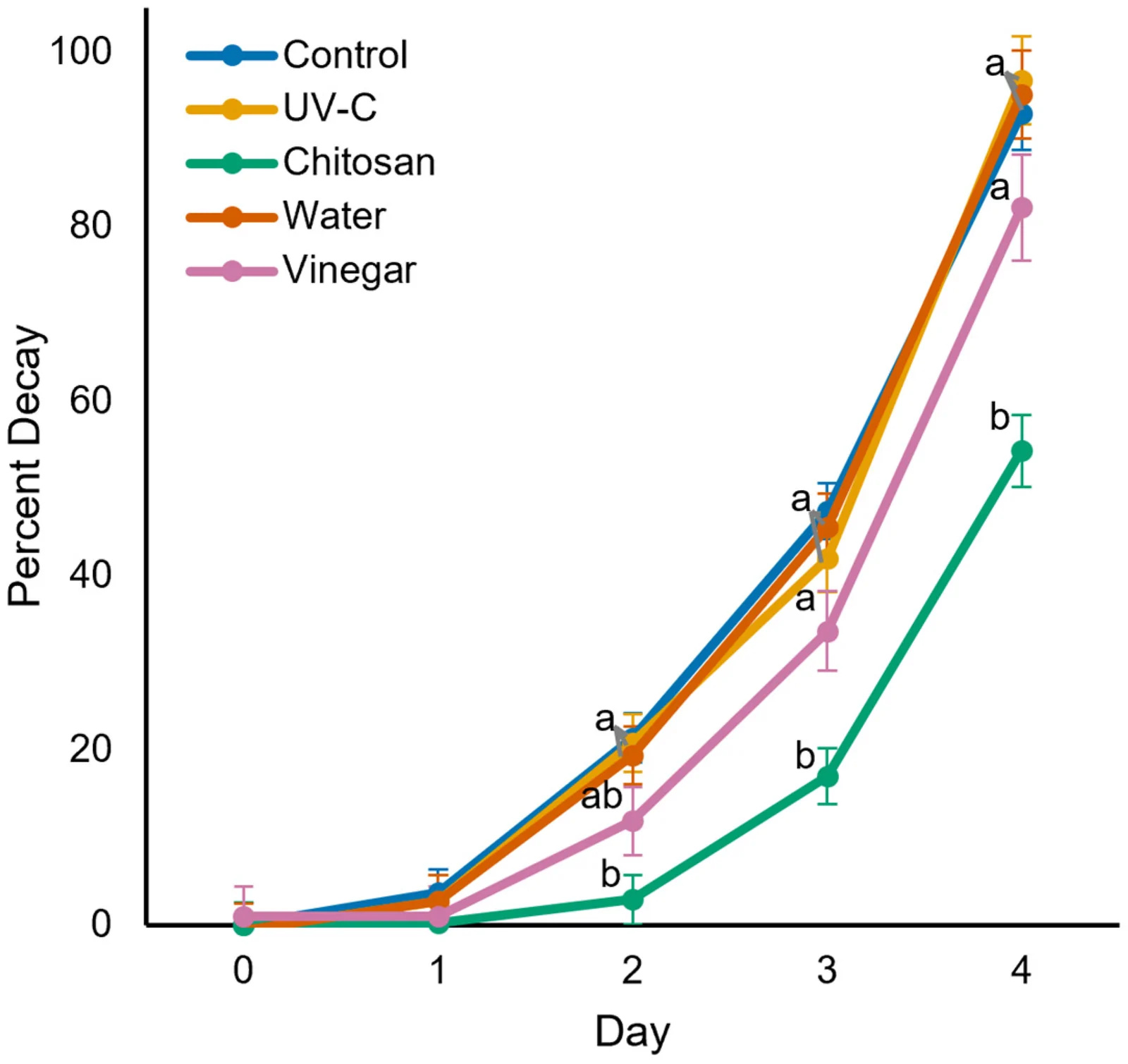
Mean decay percentage of non-treated (control), chitosan-, UV-C-, water-, and vinegar-treated strawberries at Day 0, Day 1, Day 2, Day 3, and Day 4 stored at 21 °C. Blocks 1–5 are represented; comparisons between treatments never sharing a block (e.g., UV-C vs. vinegar) are indirect. Error bars represent standard error of the mean. Effect of treatment, *p* = 0.123; effect of day, *p* < 0.001; interaction effect, *p* < 0.001. Treatments within a day that do not share a letter are significantly different (*p* < 0.05).

The overall effects of treatment and the Day × Treatment interaction were significant (*p* < 0.001, Figure 1, Supplemental Table S1), indicating that treatment changed the trajectory of strawberry decay. By Day 2, chitosan-treated strawberries showed less decay than control strawberries, but not vinegar-treated strawberries. Chitosan-treated strawberries had significantly less decay than all other treatments by Day 3. Chitosan treatment shifted the strawberry decay curve by approximately one day later.

**Figure 2:**
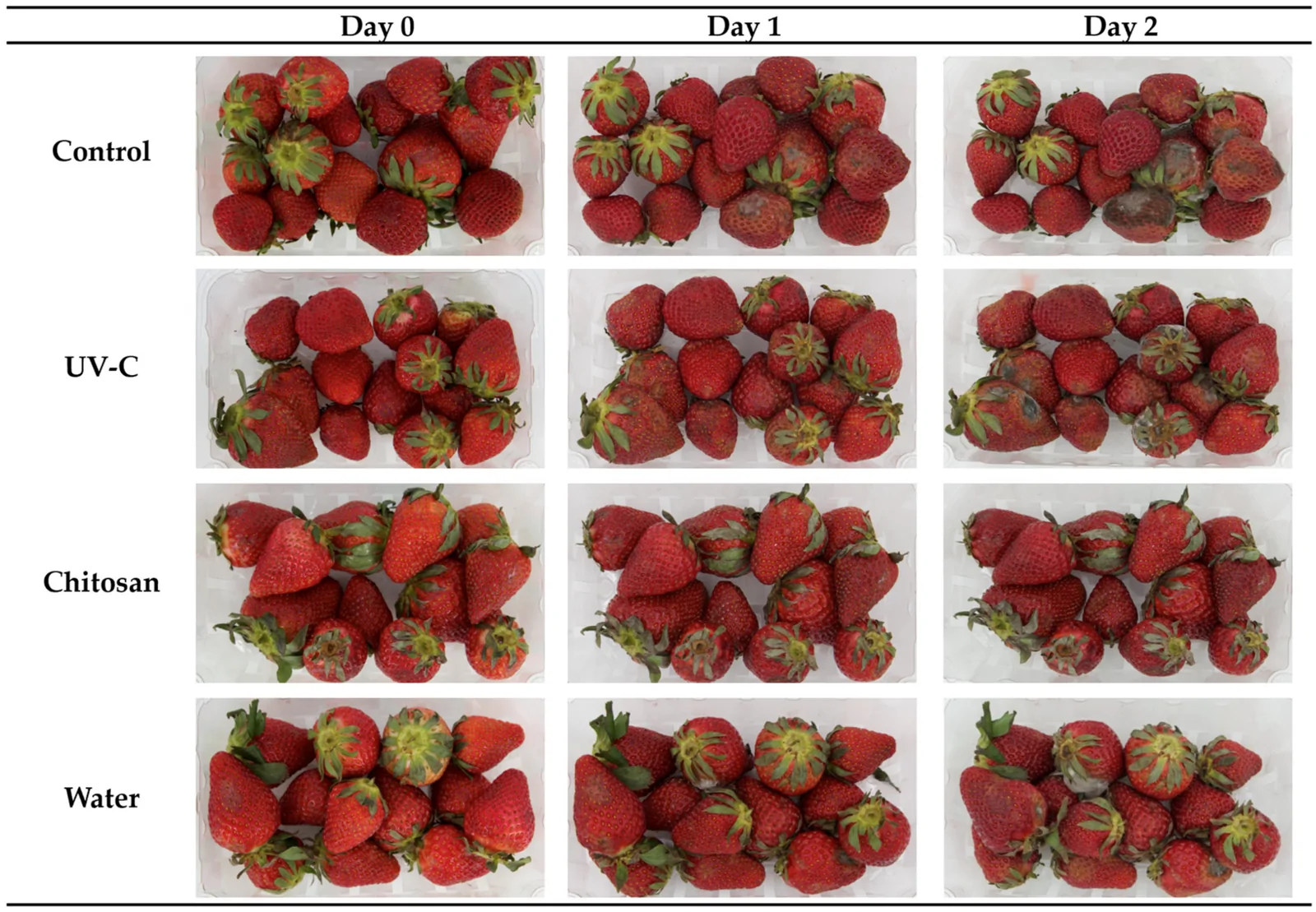
Pictures of non-treated, UV-C-, chitosan-, and water-treated strawberries on the day of treatment and Day 1 and Day 2 after treatment stored at 21 °C. Qualitative observations are consistent with the decay and microbiological enumeration of yeast and mold and represent the pictures shown to participants in the consumer acceptance survey.

Representative images of strawberries from the control, UV-C, chitosan, and water treatments are presented in Figure 2. Although these photographs are qualitative, they provide visual confirmation of the visual decay data, illustrating visible fungal growth on the control, water-, and UV-C-treated strawberries, while chitosan-treated fruit exhibited little or no visible mold under the same storage conditions.

### 3.2. Weight Loss

Storage time significantly affected strawberry weight loss (*p* < 0.001; Table 2, Supplemental Table S1). A significant Day × Treatment interaction was detected (*p* = 0.012), indicating that treatment effects varied during storage. Treatment was tested at each day; it had no effect on Day 1 (*p* = 0.616) but a significant effect on Days 2 through 4 (all *p* ≤ 0.001, Supplemental Table S1). Only vinegar, included as a control for chitosan’s acidic carrier, exhibited greater weight loss than the control, reaching 21.3% versus 14.7% by Day 4 (*p* < 0.001). Neither UV-C nor chitosan differed from the control on any day.

### 3.3. Color Analysis

Color was largely stable across time, as no main effects of day were observed for L*, a*, or b* values (*p* > 0.05, Table 3, Supplemental Table S1). Day × Treatment interaction was not significant for L* and a*, while the interaction was significant for b* (*p* = 0.013). This interaction was not retained in the model because it was driven entirely by the vinegar treatment. Vinegar had the fewest replications of any treatment, so its variation across days could not be reliably separated from random noise. When examining the main effect of day, chitosan-, water-, and UV-C-treated strawberries were darker (lower L*) than the control (effect of treatment, *p* < 0.001). Vinegar did not differ from the control in lightness. All four treatments were less yellow (lower b*) than the control (effect of treatment, *p* < 0.001). No treatment effects were observed for a* values (Table 3). Although the treatment effects on L* and b* were statistically significant, the effect sizes were small.

### 3.4. Texture Analysis

Significant texture differences were observed for day in bioyield, elasticity, and stiffness (*p* < 0.001), but not work to penetrate (*p* = 0.193, Table 4, Supplemental Table S1). Treatment effects were observed for bioyield (*p* = 0.005), work to penetrate (*p* < 0.001), and stiffness (*p* = 0.003); the Day x Treatment interaction was not significant for bioyield, stiffness, or work to penetrate, suggesting that observed effects were largely due to initial treatment and not texture-preserving effects over time. Elasticity showed a significant Day × Treatment interaction and is discussed below.

Bioyield decreased over time across all treatments, declining by approximately 15% per day (Supplemental Table S1). Averaged across all days, bioyield did not differ significantly between the control and chitosan, UV-C, and vinegar-treated strawberries. However, the strawberries treated with water exhibited significantly lower bioyield than the control treatment.

The effect of treatment on strawberry elasticity differed by day, as the Day × Treatment interaction was significant (*p* = 0.028). By Day 4, chitosan-treated strawberries were significantly less elastic than the control; UV-C and water did not significantly differ from the control (Table 4).

All fruits decreased in stiffness over time by approximately 17% per day (*p* < 0.001). There was no significant difference between each treatment and control. There was a significant difference between chitosan and UV-C, as well as chitosan and water, with chitosan retaining more stiffness on average (Table 4).

Unlike the other texture analysis outcomes, work to penetrate did not significantly change over treatment days (Table 4). Averaged across all days, vinegar, chitosan, and UV-C treatments were not significantly different from the control; only water showed significantly less work to penetrate than the control.

### 3.5. Yeast, Mold, and Fungal Testing

As seen in Figure 2, mold was visible in some strawberries, but not others, leading to variable yeast and mold counts when randomly selecting strawberries; thus, median rather than mean CFU counts are reported. Treatment had a significant effect on yeast and mold counts (*p* < 0.001, Table 5, Supplemental Table S1), with chitosan-treated strawberries showing significantly lower median CFU than all other treatments. UV-C-treated strawberries exhibited significantly higher yeast and mold counts than the control. Considering the high amount of censored data in the analysis, median values in Table 5 should be interpreted as differences in treatment magnitude rather than precise estimates. However, the observation that the majority of yeast and mold counts for chitosan-treated strawberries fell below the limits of detection is itself an informative result, regardless of the exact magnitude. Furthermore, the observed yeast and mold counts are consistent with the visual evidence shown in Figure 2.

**Figure 3:**
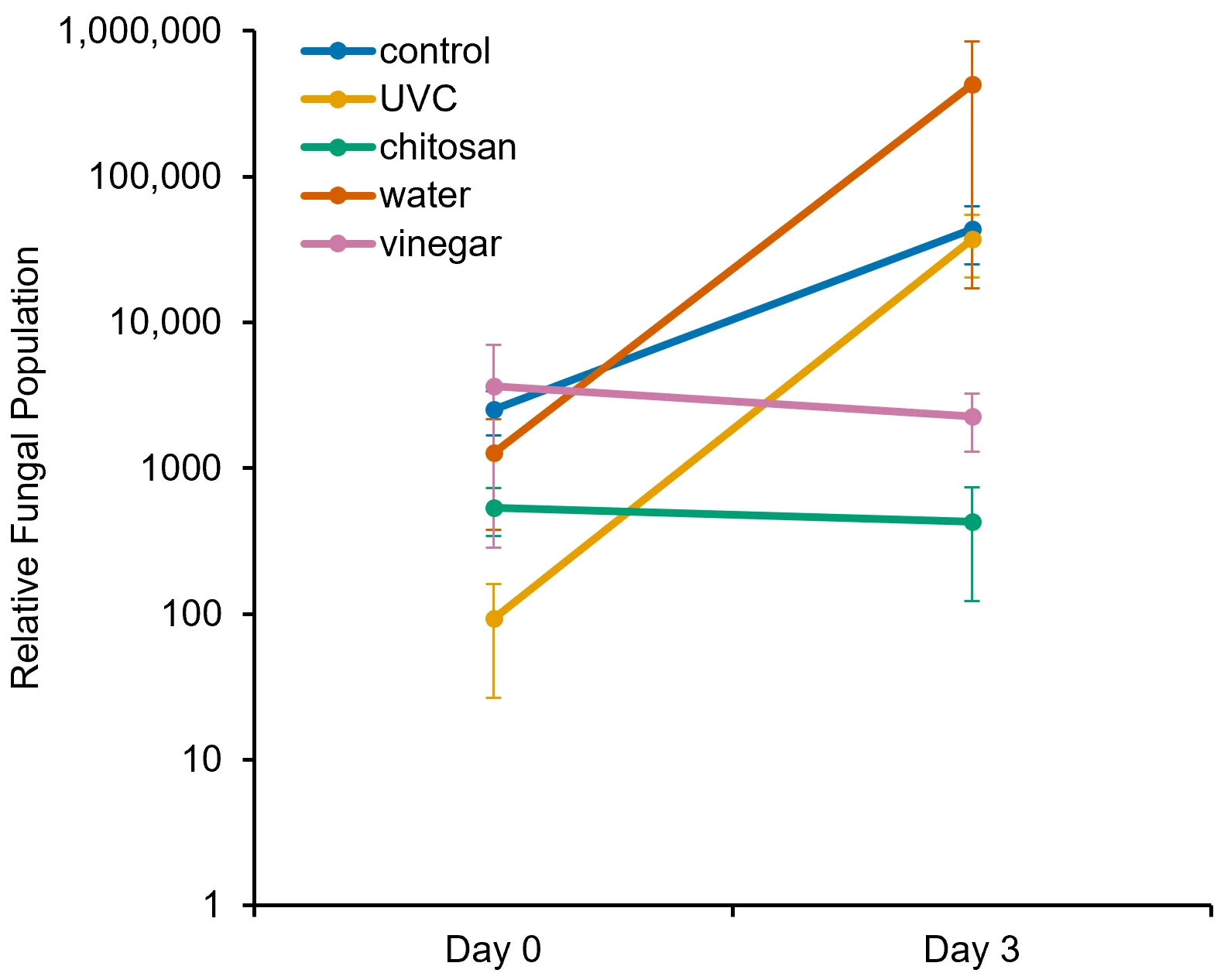
Relative abundance of fungal DNA in samples collected from strawberry surfaces as determined by qPCR from Block 6. Fungal DNA amplification (ITS) was normalized to strawberry genomic DNA amplification. Points represent mean; error bars represent standard error of the mean across 6 replicate samples stored at 21 °C. No significant effect of treatment was detected either day based on one-way ANOVA. Exploratory independent samples *t*-tests revealed UV-C- and chitosan-treated strawberries were lower than control on day 0, and that chitosan- and vinegar-treated strawberries were lower than control on Day 3 (*p* < 0.05).

No effect of treatment on Day 0 or Day 3 fungal populations was observed (F(4, 25) = 0.654, *p* = 0.626; F(4, 25) = 0.989, *p* = 0.432, respectively). The non-significant omnibus result likely reflects limited statistical power given the small sample size (*n* = 3 biological replicates per group) and elevated variability among samples. Therefore, we conducted exploratory post hoc analysis of each treatment versus the control to further test study hypotheses and treat findings as exploratory rather than confirmatory. Based on independent samples *t*-tests, amplification of fungal and strawberry DNA from samples treated with control treatments or antimicrobial treatments via qPCR suggested that antimicrobial treatments lowered fungal biomass (Figure 3). On the day of treatment, strawberries treated with chitosan or UV-C had less fungal load than control berries (*p* = 0.023 and 0.008, respectively). Three days later, chitosan-treated strawberries still had significantly reduced fungal biomass load (*p* = 0.019), but the UV-C-treated strawberries had fungal populations that recovered to the level of control strawberries. At 3 days post-treatment, the vinegar-treated berries also had significantly less fungal biomass than the control (*p* = 0.023).

### 3.6. Consumer Appearance Ratings

**Figure 4:**
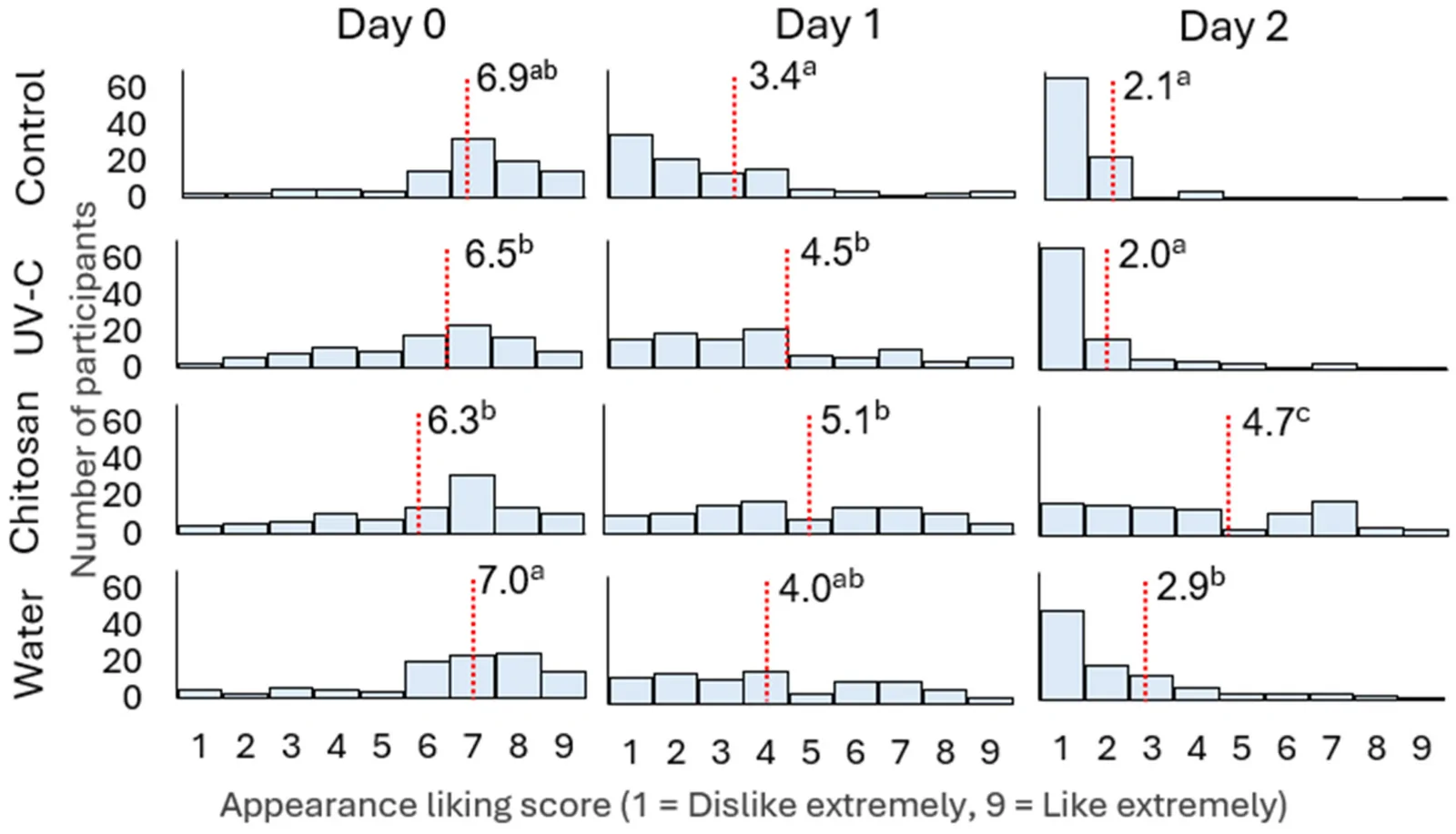
Estimated marginal means and distributions of appearance liking scores of photographs of control, UV-C-, water-, and chitosan-treated strawberries on the day of treatment and on Day 1 and Day 2 after treatment, stored at 21 °C. Number of participants who gave each score is indicated on the y-axis. Effect of treatment, *p* < 0.001; effect of day, *p* < 0.001; interaction effect, *p* < 0.001. Treatments within a day with different superscript letters are significantly different (alpha < 0.05).

We next examined the effect of day and treatment on the appearance liking of treated strawberries using an online survey. Treatment, day, and their interaction were all significant (*p* < 0.001 for each term, Supplemental Table S1, Figure 4). The appearance of strawberries from both the UV-C and chitosan treatments was liked more than the control by Day 1 (*p* < 0.001). By Day 2, the appearance of the chitosan-treated strawberries was liked more than all other treatments (*p* < 0.001). Although the mean appearance liking score of chitosan-treated strawberries on Day 2 corresponded with a rating of “neither like nor dislike”, the bimodal distribution of responses shows a divergence of accepting and non-accepting participants, as approximately 36% of participants rated these strawberries at least “like moderately” (Figure 4).

In addition to rating appearance liking, participants were also asked to endorse both positive (tasty, safe to eat, and want to eat) and negative (off-texture, spoiled, moldy, off-color) terms to describe each photo (Table 6). By Day 1, the positive terms “tasty” (chitosan only), “safe to eat” (both), and “fresh” were endorsed more frequently in treated strawberries than the control, while the negative terms, “moldy” (both) and “spoiled” (both) were endorsed less frequently than the control. By Day 2, only the picture of the chitosan-treated strawberries elicited higher endorsement of positive terms and lower endorsement of negative terms (except “off-texture”).

## 4. Discussion

In this study, we found that a solution of 1% chitosan in vinegar prolonged the shelf-life of post-retail strawberries across multiple measures, including percent decay, yeast and mold growth, fungal populations, and visual acceptance, while having minor effects on texture and color. UV-C exposure was largely ineffective. These findings extend the previous literature by examining the effect of established shelf-life extension technologies in post-retail rather than freshly harvested strawberries. Furthermore, this study pairs objective measures of quality with consumer visual acceptance data.

### 4.1. Microbial Control and Consumer Acceptance

Chitosan treatment delayed decay and lowered yeast and mold counts and maintained consumer appearance acceptability in post-retail strawberries. While UV-C likely lowered initial fungal presence immediately after treatment, it had minimal effect on final microbial growth or consumer acceptance.

Previous studies have demonstrated that chitosan coatings reduce visible mold incidence, lower yeast and mold counts, and delay decay in freshly harvested strawberries under cold-storage conditions [41,43,44]. The present study extends the previous literature by demonstrating that chitosan can also delay decay in post-retail strawberries. While both colony counts and qPCR data suggest an antimicrobial effect of vinegar, we note that the chitosan-coating lowered yeast and mold counts beyond the effect of vinegar alone, suggesting an antifungal effect for chitosan itself. The observations of yeast and mold counts in vinegar-treated strawberries should be interpreted cautiously, as many samples from both groups fell below the limit of detection and were substituted at the lower detection threshold. Regardless, the antifungal mechanism of chitosan in post-retail strawberries requires further research.

The potential impact of chitosan on fungal populations on the initial day of treatment suggests a rapid antifungal effect that was also able to persist. Chitosan may have lowered yeast and mold counts and delayed visual mold appearance in post-retail tissue through direct antifungal activity against surface fungi, including *Botrytis cinerea*, which has been shown to remain active regardless of the ripening stage of the host tissue [13,42]. While previous studies have identified the effect of chitosan in eliciting host defense responses, such as antioxidant enzyme upregulation and cell wall reinforcement observed in freshly harvested strawberries [13,42], these effects were not assessed in this study and are an area for future research. As the previous literature suggests that defense responses may be muted in advanced senescence, chitosan’s efficacy in the current study likely resulted from direct antifungal mechanisms rather than defense elicitation.

UV-C irradiation provided a measurable but transient reduction in fungal populations on post-retail strawberries. While exploratory analyses suggest that fungal DNA was reduced by UV-C treatment relative to the control, this reduction was not maintained during subsequent storage. By three days post-treatment, fungal biomass on UV-C-treated strawberries was no longer significantly different from untreated controls, suggesting recovery or regrowth of surviving fungal populations. Similarly, UV-C treatment did not significantly reduce final yeast and mold counts, visible decay incidence, or substantially improve consumer acceptance relative to untreated strawberries. Given the exploratory nature of the fungal quantitation, additional confirmatory analyses are needed to assess the effect of UV-C and chitosan on fungal populations generally and *Botrytis cinerea* specifically.

In the present study, the observed immediate reduction in fungal DNA abundance suggests that UV-C exposure exerted a direct antimicrobial effect on surface-associated fungi. However, the absence of sustained suppression indicates that this effect was insufficient to meaningfully alter spoilage progression under the storage conditions examined. Insufficient UV-C penetration to subsurface fungal contamination established prior to treatment is a likely explanation. Penetration depth is a known limitation of UV-C treatment, especially in solids [35]. Consistent with the recovery of fungal populations in UV-C-treated strawberries observed in the current study, another study observed that mycelial growth recovered after the first day of UV-C treatment at doses of 0.015 to 1.57 kJ/m^2^ (a range that includes the dose used here) and that reductions in spore germination diminished as time from treatment increased [57]. Furthermore, while doses as high as 35.1 kJ/m^2^ inactivated *Penicillium expansum* on apple surfaces, no effect on the growth of infections established in wounded tissue was observed [58]. Because those doses are far above the fluence applied here, this constraint appears to reflect physical access rather than dose. Thus, fungal contamination that has extended beneath the fruit surface in post-retail produce is likely inaccessible to UV-C, limiting the efficacy of the treatment. In addition to penetration limitations, insufficient fluence may also have contributed to a lack of UV-C effectiveness. While the dosage used in the current study was intentionally low to avoid excessive damage to aged fruit, our nominal two-sided dose of 0.079 kJ/m^2^ falls below the 0.1 to 50 kJ/m^2^ range of doses explored in postharvest UV-C research [35]. Our findings are therefore better interpreted as a test of one low fluence than as a general test of UV-C in post-retail fruit. Finally, many beneficial effects attributed to UV-C in fresh produce are associated with activation of host defense pathways. Because the strawberries used in this study had already undergone retail handling and entered a more advanced stage of senescence, physiological responsiveness to UV-C-induced defense signaling likely differs from freshly harvested fruit.

Visible mold growth, rather than perceived color or texture differences, appeared to be the primary driver of consumer acceptance, as “moldy” CATA endorsement rates were associated with larger differences in liking scores than “off-color” or “off-texture” (Supplemental Table S2). By Day 2, endorsement of “moldy” and “spoiled” was significantly lower in chitosan-treated strawberries than in control or UV-C-treated strawberries. As prior work has demonstrated the centrality of visual spoilage cues in disposal decisions at the retail and household level [5,59], findings from the present study suggest a potential impact on food waste behaviors. At 24 h post-retail, untreated control strawberries were largely unacceptable to consumers: only 4% gave them positive appearance-liking ratings, and only 10% considered them safe to eat. In contrast, chitosan-treated strawberries remained more acceptable even at 48 h post-retail, with 37% of consumers giving positive appearance-liking ratings and 28% considering them safe to eat. Together, these results suggest that most consumers would reject untreated strawberries after 24 h of room-temperature storage, whereas chitosan treatment may extend consumer acceptability by at least one additional day. Although our data suggest that a considerable segment of consumers would reject chitosan-treated strawberries by Day 2, a 1–2-day shelf-life extension for approximately one third of consumers would represent a meaningful reduction in food waste behavior. Taken together, these findings suggest that the shelf-life extending potential of chitosan can alter consumer perceptions in addition to objective indicators of quality.

### 4.2. Color

Chitosan and UV-C treatment resulted in marginally lower L* values (darker) and b* values (less yellow) than untreated controls, though the absolute differences were small (ΔL* ≤ 0.87 units; Δb* 0.96–1.56 units, Table 3). No treatment differed from the control on the a* axis. While previous studies have shown that chitosan or UV-C treatment applied to freshly harvested strawberries can slow color deterioration [27], the absence of significant color changes over time in any treatment condition demonstrates that no additional color deterioration occurred during the treatment period. Previous studies of strawberries have shown that strawberries show darker color (lower L*), stable a*, and more blue tones (lower b*) as anthocyanins are broken down by enzymes or interact with reactive oxygen species, resulting in more blue tones [43,60,61,62,63]. Although lower b* values in treated strawberries may be indicative of slowed microbial deterioration due to less browning, anthocyanin changes, and chlorophyll degradation [64,65], the small change in absolute b* values limits meaningful interpretation of the study’s findings. Furthermore, consumer acceptance data showed that “off-color” was endorsed less frequently for chitosan picture on Day 1, but there was no difference on Day 2, suggesting that changes in color were too subtle to be significant drivers of appearance liking.

### 4.3. Texture and Moisture Loss

Minimal effects of shelf-life extension treatments on texture and moisture loss in post-retail strawberries were observed, as only elasticity values were impacted by chitosan and UV-C treatments. Strawberries typically experience softening due to moisture loss and enzymatic breakdown of the cell wall, resulting in lower bioyield, stiffness, and work to penetrate and higher elasticity [49,50,66,67,68,69,70,71]. Consistent with these observations, we also observed lower bioyield and stiffness and higher elasticity, but no change in work to penetrate. While previous studies have shown that chitosan can slow water loss [22,38,72], chitosan’s slowing of water loss in the current study did not reach statistical significance, which may explain the absence of meaningful changes in texture analysis. However, chitosan appeared to mitigate the moisture loss associated with vinegar treatment: by Day 4, chitosan-treated fruit retained approximately 13% more weight than vinegar-treated fruit, consistent with previous observations that high vinegar concentrations can promote cellular damage and water loss [72,73]. These results suggest that chitosan may have exerted a protective effect in the present study.

Prior studies on freshly harvested strawberries have reported texture preservation with chitosan and UV-C under cold-storage conditions [5,22,43,74]. The absence of a comparable treatment effect here may reflect two constraints specific to this study. Post-retail fruit may have already undergone substantial cell wall degradation prior to treatment, limiting the scope for surface treatments to reverse or slow softening. In addition, room-temperature storage conditions likely accelerated cell wall degradation across all treatments relative to cold storage, potentially masking modest treatment effects on texture.

### 4.4. Strengths, Limitations, Future Directions

Several features of this study strengthen interpretation of its findings. Pairing objective quality metrics, including visible decay, yeast and mold counts, colorimetry, and texture analysis, with consumer visual acceptance data provides insight into how shelf-life extension technologies may influence food waste behaviors. Because visual cues, rather than objective measures, are a key driver of produce disposal behaviors [5], the prolonging of consumer visual acceptance of chitosan-treated strawberries suggests a potential opportunity to reduce food waste. Furthermore, the post-retail context addresses a gap in the postharvest literature, which has primarily evaluated shelf-life extension treatments applied at or immediately after harvest.

The current findings should be interpreted in light of several limitations. UV-C irradiance was not measured at the fruit surface, and the fluence reported here is a nominal estimate that does not account for shading or surface curvature. The nominal dose fell below the range of fluences explored in the postharvest UV-C literature, and we did not conduct a dose–response experiment. Exposure time was limited during preliminary testing by mold development and skin discoloration at longer exposures, but these observations were subjective and were not systematically recorded. We therefore raise the possibility that the exposure tolerated by post-retail fruit may be lower than the exposure required for efficacy only as a hypothesis for future testing. Vinegar was evaluated only in Blocks 4–5, while UV-C and water were evaluated only in Blocks 1–3; block-level effects cannot be fully disentangled from treatment effects for those comparisons. Furthermore, vinegar-treated strawberries were not included in the consumer survey; thus, whether the effects of chitosan on visual acceptance were attributable to chitosan alone or the chitosan-vinegar combination cannot be determined. However, because chitosan vs. non-treated strawberries were compared in every block, the key findings of the study regarding chitosan in vinegar solution treatment are robust. We note that the high level of censored data from the yeast and mold count limits interpretation of precise yeast and mold count estimates; however, despite the high level of censoring, a significant effect of treatment was still detected. Future studies with wider dilution ranges are needed to provide more precise estimates of yeast and mold. While room-temperature storage conditions were chosen to provide realistic context for possible household or grocery rescue conditions, these conditions accelerated deterioration, which limits direct comparison with cold-storage postharvest studies. Consumer acceptance was assessed from photographs rather than direct sensory evaluation; appearance liking and perceived safety are captured, but flavor, aroma, and in-mouth texture acceptability are not. Sample sizes for yeast and mold count data were limited by destructive sampling constraints, and several early-day samples fell below the limit of detection, requiring substitution and limiting precision of effect estimates. Finally, mechanistic variables measuring markers of senescence, including abscisic acid signaling, antioxidant enzyme activity, and cell wall enzyme activity, were not measured; mechanistic interpretations in this study are therefore inferred from the prior literature rather than directly demonstrated.

Future work could explore the efficacy of chitosan coating in various treatment or storage conditions, such as under refrigerated post-retail conditions or the use of chitosan in combination with synergistic ingredients such as essential oils [38]. Additionally, measuring biochemical defense markers including abscisic acid, antioxidant enzyme activity, and phenolic compound accumulation in post-retail versus freshly harvested tissue following treatment would directly assess the role of host defense elicitation in senescent fruit. Lastly, assessing in-person sensory acceptability or disposal behavior of chitosan-treated post-retail strawberries would determine whether the visual acceptance observed in the present study translates to meaningful changes in food waste. For example, sensory evaluation could identify whether the slightly sour taste imparted by the chitosan in vinegar solution lowers acceptability. Evaluating the cost-feasibility of chitosan treatment in grocery rescue program contexts or household applications would be needed before practical adoption could be recommended. Future studies may also explore the extent to which chitosan coatings extend to other post-retail produce.

## 5. Conclusions

Chitosan coating preserved the visual quality and consumer acceptability of post-retail strawberries more effectively than low-dose UV-C or control strawberries by approximately 1–2 days. Meanwhile, instrumental color and texture differences among treatments were generally small or inconsistent, suggesting that objective quality metrics alone may not fully capture the consumer-relevant quality differences in aged produce. It is significant to note that this work was conducted using post-retail strawberries rather than freshly harvested fruit, providing insight into interventions that may be implemented after fruit has already entered the commercial supply chain. These findings suggest a practical role for chitosan treatment in extending the window of acceptance for post-retail strawberries, with potential implications for grocery rescue programs and food waste reduction efforts. Future work should assess in-person sensory acceptability and treatment cost-feasibility and directly measure defense response activity in post-retail versus freshly harvested tissue to clarify the mechanisms underlying chitosan’s shelf-life extending potential.

## Figures and Tables

**Table 1:** Model details for statistical analyses.

Response	Model	Transformation	Fixed Effects	Random Intercepts
Weight	Linear Mixed Model	Log	Treatment, Day, Initial Weight, Treatment × Day	Block, Container within Block
Bioyield	Linear Mixed Model	Log	Treatment, Day	Block, Container within Block
Elasticity	Linear Mixed Model	None	Treatment, Day, Treatment × Day	Block, Container within Block
Penetration	Generalized Linear Mixed Model (Gamma, log link)	None	Treatment, Day	Block, Container within Block
Stiffness	Generalized Linear Mixed Model (Gamma, log link)	None	Treatment, Day	Block, Container within Block
L*	Linear Mixed Model	None	Treatment, Day	Block, Container within Block, Strawberry within Container
a*	Linear Mixed Model	None	Treatment, Day	Container within Block, Strawberry within Container
b*	Linear Mixed Model	None	Treatment, Day	Block, Container within Block, Strawberry within Container
Decay	Linear Mixed Model	None	Treatment, Day, Treatment × Day	Block, Container within Block
CFU	Interval Censored Permutation test	None	Treatment	

**Table 2:** Mean (standard deviation) initial weight per strawberry averaged within each container, and percent change from the initial weight of non-treated, UV-C-, chitosan-, water-, and vinegar-treated strawberries at Day 1, Day 2, Day 3, and Day 4 after treatment, stored at 21 °C. Blocks 1–5 are represented; comparisons between treatments that never share a block (e.g., UV-C vs. Vinegar) are indirect. Effect of treatment, *p* = 0.348; effect of day, *p* < 0.001; interaction effect, *p* = 0.012. Percentages within a day that do not share a letter are significantly different based on the model adjusted for the initial weight (*p* < 0.05, Tukey adjusted). Treatment did not significantly affect weight change on Day 1 (*p* = 0.616). Strawberries were stored at 21 °C.

	Initial Weight (g/Fruit)	Day 1 (%)	Day 2 (%)	Day 3 (%)	Day 4 (%)
Control	31.0 (7.5)	−3.6 (0.9)	−7.1 (1.3) ^a^	−10.6 (1.7) ^a^	−14.7 (2.8) ^a^
UV-C	31.9 (9.0)	−4.0 (1.3)	−8.4 (2.1) ^a^	−13.0 (2.3) ^a^	−17.3 (3.1) ^a^
Chitosan	31.7 (7.6)	−4.0 (2.3)	−5.7 (6.3) ^a^	−9.4 (6.8) ^a^	−12.9 (6.7) ^a^
Water	33.6 (10.2)	−4.8 (1.8)	−8.3 (1.8) ^ab^	−11.4 (2.4) ^a^	−17.8 (7.8) ^a^
Vinegar	34.8 (4.6)	−3.7 (6.3)	−9.7 (6.8) ^b^	−15.0 (4.6) ^b^	−21.3 (2.3) ^b^

**Table 3:** Mean (standard deviation) of L* (higher indicates lighter), a* (higher is redder), and b* (higher is more yellow) color values at Day 1, Day 2, and Day 3 after treatment. Blocks 1–5 are represented; comparisons between treatments never sharing a block (e.g., UV-C vs. vinegar) are indirect. Effects for L*, a*, b*, respectively of treatment *p* < 0.001, *p* = 0.639, *p* = 0.123, day, *p* = 0.281, *p* = 0.427, *p* = 0.965; and interaction *p* = 0.186, *p* = 0.498, *p* = 0.013. Letters in the Contrasts column compare the main effect of treatment across all sampling days; treatments that do not share a letter differ significantly (*p* < 0.05, Tukey-adjusted). “ns” indicates the treatment effect was not significant. Strawberries were stored at 21 °C.

	Treatment	Day 1	Day 2	Day 3	Contrasts
L*	Control	30.8 (4.8)	31.1 (5.3)	30.3 (5.4)	a
	UV-C	27.6 (4.1)	28.6 (5.6)	26.4 (3.2)	b
	Chitosan	29.9 (4.3)	30.9 (5.3)	29.5 (4.3)	b
	Water	27.6 (4.3)	28.7 (6.6)	26.7 (3.3)	b
	Vinegar	35.5 (3.7)	32.8 (3.3)	33.7 (3.7)	ab
a*	Control	23.2 (5.2)	27.7 (6.9)	22.3 (7.1)	ns
	UV-C	23.5 (5.8)	26.5 (6.2)	23.3 (5.7)	ns
	Chitosan	23.5 (5.2)	27.1 (6.2)	21.7 (5.8)	ns
	Water	23.8 (5.5)	26.8 (6.6)	23.4 (5.8)	ns
	Vinegar	24.2 (3.7)	27.6 (5.8)	20.8 (5.5)	ns
b*	Control	14.1 (5.3)	17.1 (6.0)	14.4 (5.0)	a
	UV-C	17.0 (3.6)	16.6 (3.7)	16.4 (3.2)	bc
	Chitosan	12.8 (5.5)	15.2 (5.4)	12.7 (5.5)	c
	Water	16.5 (3.6)	17.8 (4.1)	16.4 (3.0)	b
	Vinegar	9.2 (2.8)	13.3 (6.9)	7.7 (3.1)	bc

**Table 4:** Mean (standard deviation) bioyield (g), elasticity (mm), work to penetrate (g∙sec), and stiffness (g/sec) values at Day 1, Day 2, Day 3, and Day 4 after treatment. Blocks 1–5 are represented; comparisons between treatments never sharing a block (e.g., UV-C vs. vinegar) are indirect. UV-C-treated strawberries were beyond 90% decay in all blocks; thus, no data were collected (Not collected). Effects for bioyield, elasticity, work to penetrate, and stiffness, respectively, of treatment: *p* = 0.005, *p* = 0.032, *p* < 0.001, *p* = 0.003; day, *p* < 0.001, *p* < 0.001, *p* = 0.193, *p* < 0.001; and interaction *p* = 0.080, *p* = 0.028, *p* = 0.668, *p* = 0.311. Letters in the Contrasts column compare the main effect of treatment averaged across all sampling days; treatments that do not share a letter differ significantly (*p* < 0.05, Tukey-adjusted). For elasticity, where the Day × Treatment interaction was significant, letters are shown within each day, and the Contrasts column shows an em-dash. Strawberries were stored at 21 °C.

	Treatment	Day 1	Day 2	Day 3	Day 4	Contrasts
Bioyield (g)	Control	345.7 (208.1)	322.3 (174.6)	246.6 (151.8)	244.9 (198.3)	a
	UV-C	253.9 (158.5)	267.7 (142.3)	229.7 (113.7)	Not collected	ab
	Chitosan	389.6 (234.9)	284.6 (208.0)	278.3 (184.4)	300.9 (187.3)	a
	Water	268.0 (142.2)	246.8 (124.5)	235.1 (150.1)	177.6 (134.3)	b
	Vinegar	416.2 (222.5)	357.8 (206.4)	342.4 (247.7)	406.1 (208.7)	a
Elasticity (mm)	Control	3.8 (1.1) ^ab^	4.2 (1.1) ^a^	4.9 (1.2) ^a^	4.9 (1.2) ^a^	—
	UV-C	4.2 (1.5) ^a^	4.2 (1.0) ^a^	4.5 (1.2) ^ab^	Not collected	—
	Chitosan	4.0 (1.3) ^ab^	4.1 (1.3) ^ab^	4.5 (1.3) ^b^	4.8 (1.2) ^b^	—
	Water	3.8 (1.2) ^b^	3.7 (1.2) ^b^	4.4 (1.4) ^b^	5.4 (0.8) ^ab^	—
	Vinegar	4.5 (1.2) ^ab^	3.4 (1.0) ^a^	5.2 (1.0) ^ab^	5.3 (0.9) ^ab^	—
Work to penetrate (g∙sec)	Control	991.8 (921.7)	875.5 (491.2)	810.5 (495.6)	776.5 (604.7)	a
	UV-C	745.7 (611.7)	887.2 (533.3)	737.9 (419.2)	Not collected	ab
	Chitosan	1095.4 (813.9)	861.5 (723.0)	891.4 (652.1)	996.2 (645.0)	a
	Water	730.7 (461.2)	750.8 (509.6)	705.2 (473.7)	614.6 (463.0)	b
	Vinegar	1286.9 (792.9)	901.4 (631.1)	1178.3 (875.6)	1370.1 (725.9)	a
Stiffness	Control	66.1 (30.4)	60.4 (37.7)	41.0 (30.7)	37.6 (29.4)	ab
	UV-C	46.3 (23.7)	44.2 (22.7)	37.4 (18.4)	Not collected	b
	Chitosan	71.7 (34.0)	55.9 (34.8)	46.8 (30.6)	46.5 (29.0)	a
	Water	53.4 (26.0)	45.1 (22.4)	39.7 (25.3)	26.2 (22.6)	b
	Vinegar	68.6 (30.0)	77.3 (34.6)	51.7 (36.7)	60.4 (33.7)	ab

**Table 5:** Median yeast and mold CFU of non-treated, chitosan-, UV-C-, water-, and vinegar-treated strawberries at Day 1, Day 2, and Day 3 after treatment, stored at 21 °C. Blocks 1–5 are represented; comparisons between treatments never sharing a block (e.g., UV-C vs. vinegar) are indirect. Effect of treatment, *p* < 0.001; effect of day, *p* = 0.209; interaction effect, *p* = 0.170. Treatments that do not share a letter in the contrast column are significantly different from each other across days (*p* < 0.05), based on permutation tests after interval censoring.

Treatment	Day 1	Day 2	Day 3	Contrasts
Control	2.9 × 10^7^	6.7 × 10^7^	1.8 × 10^8^	b
UV-C	7.2 × 10^8^	5.0 × 10^8^	9.5 × 10^8^	a
Chitosan	<1 × 10^2^	<1 × 10^2^	<1 × 10^2^	d
Water	9.0 × 10^6^	8.1 × 10^7^	4.3 × 10^10^	ab
Vinegar	<1 × 10^2^	3 × 10^6^	<1 × 10^2^	c

**Table 6:** Endorsement frequency of attributes of photographs of control, UV-C-, water-, and chitosan-treated strawberries on the day of treatment and Day 1 and Day 2 after treatment. Treatments within a day with different superscript letters are significantly different (alpha < 0.05).

Day	Treatment	Tasty	Safe to Eat	Want to Eat	Fresh	Off-Color	Moldy	Spoiled	Off-Texture
Day 0	Control	36.6 ^b^	57.4 ^a^	37.6 ^a^	30.7 ^a^	21.8 ^a^	6.9 ^a^	10.9 ^a^	10.9 ^a^
	UV-C	31.7 ^b^	58.4 ^a^	38.6 ^a^	33.7 ^a^	19.8 ^a^	6.9 ^a^	7.9 ^a^	16.8 ^a^
	Chitosan	29.7 ^b^	57.4 ^a^	27.7 ^a^	30.7 ^a^	21.8 ^a^	9.9 ^a^	6.9 ^a^	16.8 ^a^
	Water	53.5 ^a^	64.4 ^a^	36.6 ^a^	43.6 ^a^	16.8 ^a^	6.9 ^a^	5.0 ^a^	11.9 ^a^
Day 1	Control	7.9 ^b^	9.9 ^b^	6.9 ^a^	5.9 ^b^	40.6 ^ab^	73.3 ^a^	61.4 ^a^	40.6 ^a^
	UV-C	13.9 ^ab^	32.7 ^a^	9.9 ^a^	12.9 ^ab^	45.5 ^a^	32.7 ^bc^	33.7 ^b^	46.5 ^a^
	Chitosan	22.8 ^a^	34.7 ^a^	15.8 ^a^	18.8 ^a^	31.7 ^b^	30.7 ^c^	30.7 ^b^	34.7 ^a^
	Water	8.9 ^b^	19.8 ^ab^	7.9 ^a^	5.9 ^b^	41.6 ^ab^	48.5 ^b^	43.6 ^b^	38.6 ^a^
Day 2	Control	3.0 ^b^	5.0 ^b^	2.0 ^bc^	2.0 ^b^	49.5 ^a^	85.1 ^a^	75.2 ^ab^	41.6 ^a^
	UV-C	2.0 ^b^	5.0 ^b^	1.0 ^c^	4.0 ^ab^	53.5 ^a^	87.1 ^a^	78.2 ^a^	46.5 ^a^
	Chitosan	17.8 ^a^	27.7 ^a^	17.8 ^a^	13.9 ^a^	40.6 ^a^	31.7 ^c^	29.7 ^c^	42.6 ^a^
	Water	7.9 ^ab^	10.9 ^b^	8.9 ^ab^	5.0 ^ab^	41.6 ^a^	67.3 ^b^	61.4 ^b^	41.6 ^a^

## Data Availability

The original data presented in the study are openly available in https://scholarsarchive.byu.edu/data/109/, accessed on 10 August 2026.

## Supplementary Materials

The following supporting information can be downloaded at: https://www.mdpi.com/article/10.3390/foods15162851/s1: Table S1: Statistical test results. Simple main effects of treatment were reported at each day for outcomes where the interaction was significant and reported in the model. Table S2. Penalty lift analysis of term endorsement data and appearance liking. Mean appearance liking scores were compared between endorsers and non-endorsers for each term.

## References

[1] UNEP (2024). Food Waste Index Report 2024. Think Eat Save: Tracking Progress to Halve Global Food Waste.

[2] ReFed (2025). From Surplus to Solutions: 2025 ReFED U.S. Food Waste Report.

[3] Riesenegger L., Hübner A. (2022). Reducing Food Waste at Retail Stores—An Explorative Study. Sustainability.

[4] Jaeger S.R., Machín L., Aschemann-Witzel J., Antúnez L., Harker F.R., Ares G. (2018). Buy, eat or discard? A case study with apples to explore fruit quality perception and food waste. Food Qual. Prefer..

[5] Schifferstein H.N.J. (2024). Changes in appearance during the spoilage process of fruits and vegetables: Implications for consumer use and disposal. Clean. Responsible Consum..

[6] Dusoruth V., Peterson H.H. (2020). Food waste tendencies: Behavioral response to cosmetic deterioration of food. PLoS ONE.

[7] Hingston S.T., Noseworthy T.J. (2020). On the epidemic of food waste: Idealized prototypes and the aversion to misshapen fruits and vegetables. Food Qual. Prefer..

[8] Palumbo M., Attolico G., Capozzi V., Cozzolino R., Corvino A., Chiara M.L.V.d., Pace B., Pelosi S., Ricci I., Romaniello R. (2022). Emerging Postharvest Technologies to Enhance the Shelf-Life of Fruit and Vegetables: An Overview. Foods.

[9] Chen D., Liu L., Gao Z., Zhao J., Yang Y., Shen Z. (2025). Preservation of Fruit Quality at Postharvest Through Plant-Based Extracts and Elicitors. Horticulturae.

[10] Khaliq G., Ali S., Gapper N., Nicola S. (2023). Editorial: Recent advances and approaches in the application of elicitors to enhance resistance mechanisms in fresh produce. Front. Sustain. Food Syst..

[11] Figueroa C.R., Jiang C.Z., Torres C.A., Fortes A.M., Alkan N. (2021). Editorial: Regulation of Fruit Ripening and Senescence. Front. Plant Sci..

[12] Barna B. (2022). Manipulation of Senescence of Plants to Improve Biotic Stress Resistance. Life.

[13] Wang S.Y., Gao H. (2013). Effect of chitosan-based edible coating on antioxidants, antioxidant enzyme system, and postharvest fruit quality of strawberries (*Fragaria* x *aranassa* Duch.). LWT-Food Sci. Technol..

[14] Zhang X., Wang M., Gan C., Ren Y., Zhao X., Yuan Z. (2023). Riboflavin application delays senescence and relieves decay in harvested strawberries during cold storage by improving antioxidant system. LWT.

[15] Petrasch S., Knapp S., Kan J., Blanco-Ulate B. (2019). Grey mould of strawberry, a devastating disease caused by the ubiquitous necrotrophic fungal pathogen *Botrytis cinerea*. Mol. Plant Pathol..

[16] Symons G.M., Chua Y.-J., Ross J.J., Quittenden L.J., Davies N.W., Reid J.B. (2012). Hormonal changes during non-climacteric ripening in strawberry. J. Exp. Bot..

[17] Li B.J., Grierson D., Shi Y., Chen K.S. (2022). Roles of abscisic acid in regulating ripening and quality of strawberry, a model non-climacteric fruit. Hortic. Res..

[18] Bai Q., Huang Y., Shen Y. (2020). The Physiological and Molecular Mechanism of Abscisic Acid in Regulation of Fleshy Fruit Ripening. Front. Plant Sci..

[19] Liu G.-S., Fu D.-Q., Zhong C.-F., Zong J., Zhou J.-H., Chang H., Wang B.-G., Wang Y.-X. (2024). Transcriptome combined with long non-coding RNA analysis reveals the potential molecular mechanism of high-CO_2_ treatment in delaying postharvest strawberry fruit ripening and senescence. Sci. Hortic..

[20] Yan B., Wang Y., Bai Y., Liu Z., Liu H., Chen X., Shen Y., Duan L. (2024). Insights into the senescent mechanisms of harvested strawberry fruit at the physiological, molecular and metabolic levels. Fruit. Res..

[21] Jyoti, Tripathi V.M. Improving Strawberry Production and Marketing Strategies: Leveraging Technology and Innovation. Proceedings of the 2024 International Conference on Cybernation and Computation (CYBERCOM).

[22] Gol N.B., Patel P.R., Rao T.V.R. (2013). Improvement of quality and shelf-life of strawberries with edible coatings enriched with chitosan. Postharvest Biol. Technol..

[23] La Scalia G., Aiello G., Miceli A., Nasca A., Alfonzo A., Settanni L. (2016). Effect of vibration on the quality of strawberry fruits caused by simulated transport. J. Food Process Eng..

[24] Shoji K., Schudel S., Onwude D., Shrivastava C., Defraeye T. (2022). Mapping the postharvest life of imported fruits from packhouse to retail stores using physics-based digital twins. Resour. Conserv. Recycl..

[25] Li H., Yin Y., Affandi F.Y., Zhong C., Schouten R.E., Woltering E.J. (2022). High CO_2_ Reduces Spoilage Caused by *Botrytis cinerea* in Strawberry Without Impairing Fruit Quality. Front. Plant Sci..

[26] Priyadarshi R., Jayakumar A., de Souza C.K., Rhim J.W., Kim J.T. (2024). Advances in strawberry postharvest preservation and packaging: A comprehensive review. Compr. Rev. Food Sci. Food Saf..

[27] Avalos-Llano K.R., Molina R.S., Sgroppo S.C. (2020). UV-C Treatment Applied Alone or Combined with Orange Juice to Improve the Bioactive Properties, Microbiological, and Sensory Quality of Fresh-Cut Strawberries. Food Bioprocess Technol..

[28] Bilgin A.B., Gunes G. (2024). Effects of Ultraviolet—C Treatment on Postharvest Physiologies and Decay of Berries: A Review. ITU J. Food Sci. Technol..

[29] (2018). Ultraviolet Radiation for the Processing and Treatment of Food.

[30] Severo J., de Oliveira I.R., Tiecher A., Chaves F.C., Rombaldi C.V. (2015). Postharvest UV-C treatment increases bioactive, ester volatile compounds and a putative allergenic protein in strawberry. LWT-Food Sci. Technol..

[31] Xu Y., Charles M.T., Luo Z., Mimee B., Tong Z., Roussel D., Rolland D., Véronneau P.Y. (2019). Preharvest UV-C treatment affected postharvest senescence and phytochemicals alternation of strawberry fruit with the possible involvement of abscisic acid regulation. Food Chem..

[32] Li D., Luo Z., Mou W., Wang Y., Ying T., Mao L. (2014). ABA and UV-C effects on quality, antioxidant capacity and anthocyanin contents of strawberry fruit (*Fragaria ananassa* Duch.). Postharvest Biol. Technol..

[33] Baka M., Mercier J., Corcuff R., Castaigne F., Arul J. (1999). Photochemical Treatment to Improve Storability of Fresh Strawberries. J. Food Sci..

[34] Nawkar G.M., Maibam P., Park J.H., Sahi V.P., Lee S.Y., Kang C.H. (2013). UV-Induced cell death in plants. Int. J. Mol. Sci..

[35] Darré M., Vicente A.R., Cisneros-Zevallos L., Artés-Hernández F. (2022). Postharvest Ultraviolet Radiation in Fruit and Vegetables: Applications and Factors Modulating Its Efficacy on Bioactive Compounds and Microbial Growth. Foods.

[36] Cho E.-R., Kim J.-Y., Oh S.-W., Kang D.-H. (2022). Inactivation of Pectobacterium carotovorum subsp. Carotovorum and Dickeya chrysanthemi on the surface of fresh produce using a 222 nm krypton–chlorine excimer lamp and 280 nm UVC light-emitting diodes. LWT.

[37] Prusky D., Romanazzi G. (2023). Induced Resistance in Fruit and Vegetables: A Host Physiological Response Limiting Postharvest Disease Development. Annu. Rev. Phytopathol..

[38] Romanazzi G., Feliziani E., Baños S.B., Sivakumar D. (2017). Shelf life extension of fresh fruit and vegetables by chitosan treatment. Crit. Rev. Food Sci. Nutr..

[39] Aranaz I., Alcántara A.R., Civera M.C., Arias C., Elorza B., Heras Caballero A., Acosta N. (2021). Chitosan: An Overview of Its Properties and Applications. Polymers.

[40] U.S. Food and Drug Administration (FDA) (2013). GRAS Notice Inventory. www.fda.gov.

[41] El-araby A., Janati W., Ullah R., Uddin N., Bari A. (2024). Antifungal efficacy of chitosan extracted from shrimp shell on strawberry (*Fragaria ananassa*) postharvest spoilage fungi. Heliyon.

[42] Zhang P., Jia H., Gong P., Sadeghnezhad E., Pang Q., Dong T., Li T., Jin H., Fang J. (2021). Chitosan induces jasmonic acid production leading to resistance of ripened fruit against *Botrytis cinerea* infection. Food Chem..

[43] Petriccione M., Mastrobuoni F., Pasquariello M.S., Zampella L., Nobis E., Capriolo G., Scortichini M. (2015). Effect of Chitosan Coating on the Postharvest Quality and Antioxidant Enzyme System Response of Strawberry Fruit during Cold Storage. Foods.

[44] Wang K., Li T., Chen S., Li Y., Rashid A. (2020). The biochemical and molecular mechanisms of softening inhibition by chitosan coating in strawberry fruit (*Fragaria* x *ananassa*) during cold storage. Sci. Hortic..

[45] Shoji K., Schudel S., Shrivastava C., Onwude D., Defraeye T. (2022). Optimizing the postharvest supply chain of imported fresh produce with physics-based digital twins. J. Food Eng..

[46] Pelletier W., Brecht J.K., Cecilia do Nascimento Nunes M., Émond J. (2011). Quality of Strawberries Shipped by Truck from California to Florida as Influenced by Postharvest Temperature Management Practices. HortTechnology.

[47] Schudel S., Shrivastava C., Rebeaud S.G., Karafka L., Shoji K., Onwude D., Defraeye T. (2022). Combining experiments and mechanistic modeling to compare ventilated packaging types for strawberries from farm to retailer. Food Packag. Shelf Life.

[48] Hernández-Muñoz P., Almenar E., Ocio M.J., Rafael G. (2006). Effect of calcium dips and chitosan coatings on postharvest life of strawberries (*Fragaria* x *ananassa*). Postharvest Biol. Technol..

[49] Blaker K.M., Plotto A., Baldwin E.A., Olmstead J.W. (2014). Correlation between sensory and instrumental measurements of standard and crisp-texture southern highbush blueberries (*Vaccinium corymbosum* L. interspecific hybrids). J. Sci. Food Agric..

[50] Rasouli M., Koushesh Saba M., Farina V. (2025). Quality changes and postharvest-losses of strawberry fruit packed in nano embedded biodegradable polyethylene films. Sci. Rep..

[51] Doyle J.J., Doyle J.L. (1987). A rapid DNA isolation procedure for small quantities of fresh leaf tissue. Phytochem. Bull..

[52] Chemidlin Prévost-Bouré N., Christen R., Dequiedt S., Mougel C., Lelièvre M., Jolivet C., Shahbazkia H.R., Guillou L., Arrouays D., Ranjard L. (2011). Validation and Application of a PCR Primer Set to Quantify Fungal Communities in the Soil Environment by Real-Time Quantitative PCR. PLoS ONE.

[53] Vainio E.J., Hantula J. (2000). Direct analysis of wood-inhabiting fungi using denaturing gradient gel electrophoresis of amplified ribosomal DNA. Mycol. Res..

[54] Chandler J., Rosenzweig C., Moss A.J., Robinson J., Litman L. (2019). Online panels in social science research: Expanding sampling methods beyond Mechanical Turk. Behav. Res. Methods.

[55] Peryam D.R., Pilgrim F.J. (1957). Hedonic scale method of measuring food preferences. Food Technol..

[56] Homez-Jara A., He Y., Duizer L., Kebede B., Osorio A.E., Corradini M.G. (2026). Consumer Perceptions of Food Deterioration: White Mushrooms as a Case Study. J. Food Sci..

[57] Hahlbohm A., Struck C., de Mol F., Strehlow B. (2025). UV-C treatment has a higher efficacy on reducing germination of spores rather than mycelium growth. Eur. J. Plant Pathol..

[58] Nicolau-Lapeña I., Rodríguez-Bencomo J.J., Colás-Medà P., Viñas I., Sanchis V., Alegre I. (2023). Ultraviolet Applications to Control Patulin Produced by Penicillium expansum CMP-1 in Apple Products and Study of Further Patulin Degradation Products Formation and Toxicity. Food Bioprocess Technol..

[59] Coulthard H., Abdullahi N., Bell K., Noon E. (2022). Understanding disgust-based food rejection in picky and non-picky eaters: Willingness to touch and taste familiar foods with changes. Food Qual. Prefer..

[60] Ladika G., Strati I.F., Tsiaka T., Cavouras D., Sinanoglou V.J. (2024). On the Assessment of Strawberries’ Shelf-Life and Quality, Based on Image Analysis, Physicochemical Methods, and Chemometrics. Foods.

[61] Teribia N., Buvé C., Bonerz D., Aschoff J., Hendrickx M.E., Van Loey A.M. (2021). Acidification of Strawberry Puree Affects Color and Volatile Characteristics during Storage. ACS Food Sci. Technol..

[62] Keutgen A.J., Pawelzik E. (2007). Modifications of Strawberry Fruit Antioxidant Pools and Fruit Quality under NaCl Stress. J. Agric. Food Chem..

[63] Person T., Tobler E., Schouten R., Lukasse L., Defraeye T. (2025). Multimetric assessment of packaged strawberry quality under controlled supply chain temperatures. Food Packag. Shelf Life.

[64] Verbeyst L., Oey I., Van der Plancken I., Hendrickx M., Van Loey A. (2010). Kinetic study on the thermal and pressure degradation of anthocyanins in strawberries. Food Chem..

[65] Nunes M.C.d.N., Brecht J.K., Morais A.M.M.B., Sargent S.A. (2005). Possible Influences of Water Loss and Polyphenol Oxidase Activity on Anthocyanin Content and Discoloration in Fresh Ripe Strawberry (cv. Oso Grande) During Storage at 1 °C. J. Food Sci..

[66] Moya-León M.A., Mattus-Araya E., Herrera R. (2019). Molecular Events Occurring During Softening of Strawberry Fruit. Front. Plant Sci..

[67] Jiang J., Zhang M., Devahastin S., Yu D. (2021). Effect of ultrasound-assisted osmotic dehydration pretreatments on drying and quality characteristics of pulsed fluidized bed microwave freeze-dried strawberries. LWT.

[68] Cao S., Hu Z., Pang B., Wang H., Xie H., Wu F. (2010). Effect of ultrasound treatment on fruit decay and quality maintenance in strawberry after harvest. Food Control.

[69] An X., Li Z., Wegner G., Zude-Sasse M. (2023). Effect of cell size distribution on mechanical properties of strawberry fruit tissue. Food Res. Int..

[70] An X., Li Z., Zude-Sasse M., Tchuenbou-Magaia F., Yang Y. (2020). Characterization of textural failure mechanics of strawberry fruit. J. Food Eng..

[71] Del Olmo I., Romero I., Alvarez M.D., Tarradas R., Sanchez-Ballesta M.T., Escribano M.I., Merodio C. (2022). Transcriptomic analysis of CO_2_-treated strawberries (*Fragaria vesca*) with enhanced resistance to softening and oxidative stress at consumption. Front. Plant Sci..

[72] Ochoa-Velasco C.E., Guerrero-Beltrán J.Á. (2014). Postharvest quality of peeled prickly pear fruit treated with acetic acid and chitosan. Postharvest Biol. Technol..

[73] Hassenberg K., Geyer M., Ammon C., Herppich W. (2011). Physico-chemical and sensory evaluation of strawberries after acetic acid vapour treatment. Eur. J. Hortic. Sci..

[74] Jin P., Wang H., Zhang Y., Huang Y., Wang L., Zheng Y. (2017). UV-C enhances resistance against gray mold decay caused by *Botrytis cinerea* in strawberry fruit. Sci. Hortic..

