# Supplementary material for: Post-Retail Application of Chitosan Coating and UV-C Irradiation: Effects on Strawberry Decay, Fungal Spoilage, and Consumer Acceptance
Source: Foods. 2026 Aug 15;15(16):2851. doi: 10.3390/foods15162851 (PMC13512793; doi:10.3390/foods15162851)
Supplement: Supplementary file 1 [file foods-15-02851-s001.zip › foods-4432414-supplementary.pdf]

Supplemental Table S1. Statistical test results. Simple main effects of treatment were reported at each day for outcomes where the interaction was significant and reported in the model.

Outcome	Treatment	Day	Treatment × Day
Decay	$\chi^2(4) = 448.2$, $p = 0.123$	$\chi^2(4) = 448.2$, $p < 0.001$	$\chi^2(16) = 112.9$, $p < 0.001$
Day 1	All values 0, test meaningless	—	—
Day 2	$\chi^2(4) = 13.59$, $p = 0.009$	—	—
Day 3	$\chi^2(4) = 25.93$, $p < 0.001$	—	—
Day 4	$\chi^2(4) =19.19$, $p < 0.001$	—	—
Weight	$F(4, 150.0) = 1.12$, $p = 0.348$	$F(1, 158.4) = 211.26$, $p < 0.001$	$\chi^2(4) = 12.79$, $p = 0.012$
Day 1	$F(4, 151.4) = 0.67$, $p = 0.616$	—	—
Day 2	$F(4, 151.4) = 4.78$, $p = 0.001$	—	—
Day 3	$F(4, 151.2) = 7.38$, $p < 0.001$	—	—
Day 4	$F(4, 151.2) = 6.35$, $p < 0.001$	—	—
Bioyield	$F(4, 912.0) = 3.71$, $p = 0.005$	$F(1, 13.2) = 49.68$, $p < 0.001$	$\chi^2(4) = 8.35$, $p = 0.080$
Elasticity	$F(4, 830.5) = 2.66$, $p = 0.032$	$F(1, 15.9) = 17.02$, $p < 0.001$	$\chi^2(4) = 10.90$, $p = 0.028$
Day 1	$F(4, 899.8) = 2.88$, $p = 0.022$	—	—
Day 2	$F(4, 902.6) = 3.53$, $p = 0.007$	—	—
Day 3	$F(4, 810.0) = 3.51$, $p = 0.008$	—	—
Day 4	$F(4, 725.4) = 3.20$, $p = 0.013$	—	—
Work to penetrate	$\chi^2(4) = 18.72$, $p < 0.001$	$\chi^2(1) = 1.70$, $p = 0.193$	$\chi^2(4) = 2.37$, $p = 0.668$
Stiffness	$\chi^2(4) = 15.81$, $p = 0.003$	$\chi^2(1) = 56.89$, $p < 0.001$	$\chi^2(4) = 4.78$, $p = 0.311$
L*	$F(4, 2283.2) = 5.04$, $p < 0.001$	$F(1, 11.3) = 1.28$, $p = 0.281$	$\chi^2(4) = 6.18$, $p = 0.186$
a*	$F(4, 2281.0) = 0.63$, $p = 0.639$	$F(1, 15.2) = 0.67$, $p = 0.427$	$\chi^2(4) = 3.37$, $p = 0.498$
b*	$F(4, 2296.8) = 22.48$, $p < 0.001$	$F(1, 11.2) = 0.00$, $p = 0.965$	$\chi^2(4) = 12.64$, $p = 0.013$
Yeast and mold	$\chi^2(4) = 164$, $p < 0.001$	$\chi^2(2) = 1.26$, $p = 0.209$	$\chi^2(10) = 14$, $p = 0.170$
Appearance liking	$F(2.64, 263.63) = 34.83$, $p < 0.001$	$F(1.78, 179.22) = 289.16$, $p < 0.001$	$F(6, 600) = 29.53$, $p < 0.001$
Day 0	$F(3, 98) = 5.57$, $p = 0.001$	—	—
Day 1	$F(3, 98) = 20.53$, $p < 0.001$	—	—
Day 2	$F(3, 98) = 41.61$, $p = 0.001$	—	—

Weight also included initial weight as a covariate ($F(1, 122.1) = 734.38$, $p < 0.001$).

The Greenhouse-Geisser correction was used to calculate values for Appearance liking due to violation of sphericity.

Main effects tested by Type III ANOVA (F with Satterthwaite denominator degrees of freedom for linear mixed models; Wald χ^2 for generalized linear mixed models). Day × treatment interactions tested by likelihood ratio test comparing nested models fitted by maximum likelihood.

Supplemental Table S2. Penalty lift analysis of term endorsement data and appearance liking. Mean appearance liking scores were compared between endorsers and non-endorsers for each term.

Attribute	Mean Liking (Checked)	Mean Liking (Unchecked)	Liking Difference	# Checked	# Unchecked
Tasty	8.00	3.80	+4.21	236	964
Safe to eat	7.07	3.48	+3.59	383	817
Want to eat	8.05	3.89	+4.16	212	988
Fresh	8.14	3.88	+4.26	209	991
Off Color	3.03	5.51	−2.49	429	771
Moldy	2.21	6.27	−4.06	486	714
Spoiled	2.14	6.08	−3.94	442	758
Off texture	2.91	5.46	−2.54	393	807
